# Positive selection at codon 38 of the human *KCNE1 *(= *minK*) gene and sporadic absence of 38Ser-coding mRNAs in Gly38Ser heterozygotes

**DOI:** 10.1186/1471-2148-9-188

**Published:** 2009-08-06

**Authors:** Holger Herlyn, Ulrich Zechner, Franz Oswald, Arne Pfeufer, Hans Zischler, Thomas Haaf

**Affiliations:** 1 Institute of Anthropology, Johannes Gutenberg University, 55099 Mainz, Germany; 2 Institute of Human Genetics, Johannes Gutenberg University, 55131 Mainz, Germany; 3 Department of Internal Medicine I, University of Ulm, Robert-Koch-Str. 8, 89081 Ulm, Germany; 4 Institute of Human Genetics (IHG), Technical University of Munich, Klinikum rechts der Isar, 81675 Munich, Germany

## Abstract

**Background:**

KCNE1 represents the regulatory beta-subunit of the slowly activating delayed rectifier potassium channel (IKs). Variants of KCNE1 have repeatedly been linked to the long-QT syndrome (LQTS), a disorder which predisposes to deafness, ventricular tachyarrhythmia, syncope, and sudden cardiac death.

**Results:**

We here analyze the evolution of the common Gly38Ser variant (rs1805127), using genomic DNAs, complementary DNAs, and HEK293-expressed variants of altogether 19 mammalian species. The between species comparison reveals that the human-specific Gly38Ser polymorphism evolved under strong positive Darwinian selection, probably in adaptation to specific challenges in the fine-tuning of IKs channels. The involved amino acid exchanges (Asp > Gly, Gly > Ser) are moderately radical and do not induce apparent changes in posttranslational modification. According to population genetic analyses (HapMap phase II) a heterozygote advantage accounts for the maintenance of the Gly38Ser polymorphism in humans. On the other hand, the expression of the 38Ser allele seems to be disadvantageous under certain conditions, as suggested by the sporadic deficiency of 38Ser-coding mRNAs in heterozygote Central Europeans and the depletion of homozygotes 38Ser in the Yoruban sample.

**Conclusion:**

We speculate that individual differences in genomic imprinting or genomic recoding might have contributed to conflicting results of recent association studies between Gly38Ser polymorphism and QT phenotype. The findings thus highlight the relevance of mRNA data in future association studies of genotypes and clinical disorders. To the best of our knowledge, they moreover provide first time evidence for a unique pattern; i.e. coincidence of positive Darwinian selection and polymorphism with a sporadically suppressed expression of one allele.

## Background

The potassium voltage-gated channel, Isk-related family, member 1 (*KCNE1*)-gene is located on chromosome 21q22.12 and stretches over three exons, with the coding 381 bp being confined to the third and last exon [1]. The encoded protein (KCNE1, also termed minK) is a single-span membrane protein of about 14.4 kDa that coassembles with KCNQ1 (potassium voltage-gated channel, KQT-like subfamily, member 1), thus forming the slowly activating delayed rectifier potassium (IKs) channel of heart muscle, inner ear, and a variety of other tissues [2-4]. Upon docking of KCNE1 to KCNQ1, activation and deactivation kinetics of IKs channels are slowed down by nearly two orders of magnitude [5]. This renders KCNE1 an interesting candidate for studying the causes of cardiovascular diseases and congenital as well as aquired hearing loss. Particularly, the inherited long-QT syndrome (LQTS) has repeatedly been linked to polymorphisms of KCNE1 [e.g. [6-8]]. LQTS occurs with or without accompanying deafness and is characterized by delayed ventricular repolarization, visible as a prolonged electrocardiographic QT interval. The dysfunction predisposes patients to ventricular tachyarrhythmia, syncope, and sudden cardiac death. As sudden death may occur with the first syncope and LQTS diagnosis based solely on electrocardiography is neither sensitive nor specific enough, it is of outmost importance to screen subjects at risk for a genetic predisposition to LQTS.

Several population-wide screens have recently been carried out to check for the relevance of the supposed association between genomic *KCNE1 *variants and QT length. Two of these studies identified QT-modifying single nucleotide polymorphisms (SNPs) in non-coding regions of the *KCNE1 *locus, in particular in intron 2 [9] and in a putative promoter region [10]. Family based association analysis showed a significant association between QT length and an A/G-SNP at nucleotide position 112 (A112G; rs1805127) that codes for a glycine/serine polymorphism at codon 38 (Gly38Ser) [11]. The same SNP is undisputably associated with mortality and T-wave alternans in electrocardiograms [12] as well as with high noise susceptibility and noise-induced hearing loss [13]. On the other hand, an association between Gly38Ser and QT length has been questioned by other association studies [14-16]. Different genetic backgrounds of the studied populations have been discussed to account for the conflicting results. For example, *KCNE1 *may be in linkage disequilibrium to adjacent causal variants only in some ethnic groups, but not in others [15]. However, since all available association studies exclusively used genomic information, some additional factors might have had a confounding effect on the results of association studies. Firstly, one of both alleles might have been silenced in some individuals, due to genomic imprinting or null mutations in the regulatory region. Secondly, genomic recoding at the mRNA level might have contributed to the observed patterns. According to this, an adenosine deaminase acting on RNA (ADAR) might sporadically have recoded the adenosine at *KCNE1 *codon 38 (AGU) to inosine (IGU), which is equivalent to guanosine (GGU) for the translational machinery [17]. To pick up this thought we compared allele frequencies of genomic (gDNAs) and complementary DNAs (cDNAs) from a total of 43 human individuals. Furthermore to get an idea about the selective forces behind *KCNE1 *evolution we analyzed the available human population genetic data (HapMap phase II) as well as orthologs (gDNAs and cDNAs) from 18 non-human mammals (Eutheria).

## Results

### Genotyping of human samples and allele frequencies in the gDNA/cDNA comparison

Direct sequencing of KCNE1-coding gDNAs revealed that 19 out of 29 lymphoblastoid cell lines were heterozygous for Gly38Ser, while six and four cell lines were homozygote for 38Ser and 38Gly, respectively. *KCNE1 *sequencing of gDNAs of 14 brain samples (frontal cortices) added another five heterozygotes Gly38Ser, four homozygotes 38Ser, and five homozygotes 38Gly. All genotypes were confirmed once (brain samples) and twice (cell lines) using a reverse pyrosequencing assay (Tables 1 and 2). We take the wide agreement of the results from direct sequencing and pyrosequencing and particularly the exclusive detection of one allele in homozygotes as evidence for the reliability of the present reverse pyrosequencing assay. Twofold parallel pyrosequencing of cDNAs coding for KCNE1 widely reflected the patterns described for gDNAs (Tables 1 and 2): heterozygotes displayed an about 50:50 distribution of 38Gly- and 38Ser-coding alleles. Moreover, when homozygotes had been analysed, the pyrosequencing assay revealed an extensive (mostly absolute) excess of either the 38Gly- or the 38Ser-coding allele. In contrast to the general agreement of the allele frequencies inferred from gDNAs and cDNAs, we found exclusively 38Gly-coding alleles in the cDNAs of three individuals (cell lines 24 and 26; brain sample 11) that had been genotyped as heterozygotes Gly38Ser. Brain sample 11 was conspicuous on the gDNA level, too: though clearly heterozygous, pyrosequencing persistently detected less 38Ser- (12–22.2%) than 38Gly-coding alleles (88–77.8%) in a total of eight parallel measurements.

**Table 1:** Frequencies of 38Ser- and 38Gly-coding *KCNE1 *alleles (%112A/%112G) in gDNAs and cDNAs of lymphoblastoid cell lines, as inferred by pyrosequencing

Lymphoblastoid cell line	gDNA (%112A/%112G)		cDNA (%112A/%112G)	
	1st Run	2nd Run	1st Run	2nd Run
1	56.4/43.6	54.7/45.3	54.7/45.3	59.8/50.2
2	54.7/45.3	52.7/47.3	40.6/59.4	59.2/40.8
3	100.0/0.0	100.0/0.0	100.0/0.0	100.0/0.0
4	48.7/51.3	46.8/53.2	39.5/60.5	45.1/54.9
5	56.6/43.4	57.2/42.8	61.6/38.4	54.2/45.8
6	100.0/0.0	100.0/0.0	100.0/0.0	100.0/0.0
7	57.5/42.5	53.0/47.0	60.8/39.2	56.2/43.8
8	56.5/43.5	55.3/44.7	58.3/41.7	59.4/40.6
9	0.0/100.0	0.0/100.0	0.0/100.0	0.0/100.0
10	0.0/100.0	0.0/100.0	0.0/100.0	0.0/100.0
11	0.0/100.0	0.0/100.0	0.0/100.0	5.9/94.1
12	0.0/100.0	0.0/100.0	0.0/100.0	0.0/100.0
13	53.1/46.9	55.2/44.8	60.0/40.0	55.1/44.9
14	56.3/43.7	54.9/45.1	45.9/54.1	62.5/37.5
15	56.7/43.3	55.7/44.3	59.3/40.7	69.2/30.8
16	56.0/44.0	54.9/45.1	59.3/40.7	53.1/46.9
17	57.6/42.4	51.7/48.3	63.6/36.4	56.5/43.5
18	54.7/45.3	50.1/49.9	60.0/40.0	54.6/45.4
19	52.9/47.1	56.5/43.5	51.2/48.8	51.5/48.5
20	54.4/45.6	52.8/47.2	52.7/47.3	51.4/48.6
21	100.0/0.0	100.0/0.0	96.6/3.4	100.0/0.0
22	100.0/0.0	100.0/0.0	100.0/0.0	100.0/0.0
23	56.2/43.8	56.0/44.0	62.6/37.4	52.2/47.8
**24**	**55.0/45.0**	**53.2/46.8**	**0.0/100.0**	**0.0/100.0**
25	100.0/0.0	100.0/0.0	100.0/0.0	96.8/3.2
**26**	**54.1/45.9**	**54.7/45.3**	**0.0/100.0**	**0.0/100.0**
27	100.0/0.0	100.0/0.0	100.0/0.0	97.1/2.9
28	49.6/50.4	52.0/48.0	59.7/40.3	59.3/40.7
29	52.3/47.7	50.5/49.5	52.1/47.9	48.4/51.6

Lymphoblastoid cell lines with an absolute depletion of 38Ser-coding alleles (112A) at the cDNA level are highlighted with bold.

**Table 2:** Frequencies of 38Ser- and 38Gly-coding *KCNE1 *alleles (%112A/%112G) in gDNAs and cDNAs of brain samples (frontal cortex), as inferred by pyrosequencing

Brain sample	gDNA (%112A/%112G)	cDNA (%112A/%112G)	
		1st Run	2nd Run
1	0.0/100.0	-	-
2	0.0/100.0	-	-
3	0.0/100.0	-	-
4	100.0/0.0	100.0/0.0	100.0/0.0
5	51.8/48.2	60.0/40.0	50.1/49.9
6	0.0/100.0	-	-
7	0.0/100.0	-	-
8	52.5/47.5	52.6/47.4	53.6/46.4
9	100.0/0.0	100.0/0.0	100.0/0.0
10	100.0/0.0	100.0/0.0	100.0/0.0
**11**	22.2/77.8*	**0.0/100.0**	**0.0/100.0**
12	61.8/38.2	48.1/51.9	56.4/43.6
13	100.0/0.0	100.0/0.0	100.0/0.0
14	54.5/45.5	59.6/40.4	60.0/40.0

*The genotype of brain sample 11 has been verified by a total of eight parallel measurements (not shown). It is marked with bold as it exerts an absolute depletion of 38Ser-coding alleles (112A) at the cDNA level.

We additionally pyrosequenced the highly heterozygous A/G-SNP rs11254413 of the housekeeping gene tRNA aspartic acid methyltransferase 1 (*TRDMT1*). Based on gDNAs and cDNAs we found no evidence for a complete depletion of one allele in the cDNAs of 18 heterozygous lymphoblastoid cell lines. Instead, all heterozygotes were confirmed on the cDNA level. This suggests that neither RNA extraction nor specific conditions of some of the samples affected the allele quantification of the *KCNE1*-SNP rs1805127.

### Population genetic analyses of HapMap phase II data

As can be seen from Table 3, the Gly38Ser polymorphism is present not only in our own sample, but also in the genepools of those populations that had been genotyped in the course of phase II of the HapMap project (Central Europeans, Han-Chinese from Beijing, Japanese from Tokyo, Yoruba from Ibadan/Nigeria). Of particular interest is the finding of both alleles in sub-Saharan Yoruba, as this population is commonly regarded as the deepest coalescing among the sampled ones (see e.g. [18]). Thereafter, it is very likely that the A/G-SNP at *KCNE1 *codon 38 was already present before the divergence of modern humans. Table 3 moreover illustrates a general trend for selection in favour of heterozygotes: compared to the expectation, there are persistently less observed homozygotes (38Ser, 38Gly) and more observed heterozygotes (Gly38Ser) in Central Europeans, Han Chinese, and Yoruba. Consequently, Fstat calculates a negative fixation index, Fis, for these samples. In case of the Yoruban sample, homozygotes 38Ser are even absent, though 11 homozygotes 38Ser are to be expected given the allele frequencies. The Yoruban sample is also specific in that probability test statistic (Fstat) indicates a significant (*p*-value = 0.003) heterozygote excess exclusively in this sample, even when the *p*-value has been adjusted for multiple testing (strict Bonferroni correction). Finally, it is worth to note that Japanese from Tokyo represent the only exception from the general trend for selection in favour of heterozygotes: expected and observed genotype counts are the same with respect to homozygotes and nearly identical regarding the heterozygotes. We expect that a future extension of the HapMap dataset will change some details of the picture outlined above. For instance, it appears probable to us that additional data on the Yoruban population will contribute homozygotes 38Ser. It may moreover happen that the reported extent of heterozygote excess in Central Europeans, Han Chinese, and Yoruba will change with additional data. However, we are confident that the general trends inferred from the present dataset (heterozygote excess in the one or other population) will be confirmed in the future.

**Table 3:** Population genetic analyses of the A112G-SNP (rs1805127) encoding the Gly38Ser polymorphism of KCNE1

HapMap-sample	Genotype counts			Allele frequency		Test statistic	
	AA	GA	GG				
(number of individuals)	Homozygotes 38Ser	Heterozygotes Gly38Ser	Homozygotes 38Gly	A	G	Fis	*p-*value

Central Euro-peans (120)	O = 14 E = 18	O = 64 E = 57	O = 42 E = 46	0.383	0.617	-0.124	0.110
Han Chinese, Beijing (90)	O = 12 E = 14	O = 48 E = 43	O = 30 E = 32	0.400	0.600	-0.106	0.223
Japanese, Tokyo (88)	O = 8 E = 8	O = 36 E = 37	O = 44 E = 44	0.295	0.705	+0.023	0.660
Yoruba, Nigeria (120)	O = 0 E = 11	O = 72 E = 51	O = 48 E = 59	0.300	0.700	-0.425	**0.003**

Comparison of the observed (O) and expected (E) genotype counts reveals a general trend for less observed homozygotes and more observed heterozygotes than expected. In case of the Yoruban sample, the heterozygote excess is even significant (see fixation index, Fis, and probability for deviation from Hardy-Weinberg equilibrium, *p*-value).

### Evolution of the *KCNE1 *gene in eutherian mammals

The gDNAs and cDNAs of the 18 non-human species either encode aspartate (Asp) or glutamate (Glu) at the codon position homolog to codon 38 in human *KCNE1*. There was no evidence for a polymorphism of the codon under scrutiny in any of the non-human species for which SNP data were available at the time of the study (chimpanzee, Bornean orangutan, house mouse, Norway rat, and dog). Neither did databank mining reveal any hint for genomic recoding of this particular codon in the non-human sample, as indicated by identical triplets in the gDNA/cDNA comparison in Bornean orangutan, house mouse, Norway rat, guinea pig, European rabbit, cattle, and cat (see Table 4 for scientific species names and accession numbers).

**Table 4:** Species and *KCNE1 *accession numbers in the order of their appearence in Figure 1

Scientific name (common name)	Accession numbers (GenBank and ENSEMBL) of gDNAs (g) and cDNAs (c)
*Homo sapiens*, 38Ser variant (human)	EF514881 (g, ne, pa)
*H. sapiens*, 38Ser variant (human)	EF514882 (c, ne)
*H. sapiens*, 38Gly variant (human)	EF514883 (c, ne)
*H. sapiens*, 38Gly variant (human)	EF514884 (g, ne, pa)
*Pan troglodytes *(chimpanzee)	**ENSPTRT00000025885 **(g, pa)
*Pongo pygmaeus *(Bornean orangutan)	ENSPPYT00000013220 (g), EF514885 (c, ne, pa)
*Pongo abelii *(Sumatran orangutan)	NM_001132604 (c, pa), CR854432 (c)
*Symphalangus syndactylus *(siamang)	EF514886 (c, ne, pa)
*Macaca fascicularis *(crab-eating macaque)	EF514887 (c, ne, pa)
*Macaca mulatta *(rhesus macaque)	**ENSMMUT00000042620 **(g, pa)
*Presbytis cristata *(silvered leaf monkey)	EF514888 (c, ne, pa)
*Mus musculus *(house mouse)	ENSMUST00000051705 (g), X60457 (c, pa), NM_008424 (c), BX518225 (c), BY708822 (c), AI956381 (c), BB613272 (c), AJ507048 (c), BF540248 (c), AA667912 (c), AA667785 (c), AK028907 (c), BC094276 (c)
*Rattus norvegicus *(Norway rat)	ENSRNOT00000002717 (g), NM_012973 (c, pa), M36461 (c), BQ194830 (c), CX569446 (c)
*Cavia porcellus *(guinea pig)	ENSCPOT00000011328 (g), L20462 (c, pa)
*Oryctolagus cuniculus *(European rabbit)	L41659 (g, pa), EU008571 (c), EU008572 (c), NM_001109822 (c)
*Ochotona princeps *(pika)	**ENSOPRT00000007254 **(g, pa)
*Sus scrofa *(pig)	NM_214165 (c, pa)
*Bos taurus *(cattle)	ENSBTAT00000001530 (g), NM_001077977 (c, pa), BE486735 (c), DV843524 (c), CK849850 (c)
*Equus caballus *(horse)	**ENSECAT00000004027 **(g, pa)
*Felis catus *(cat)	ENSFCAT00000006761 (g), NM_001009206 (c, pa)
*Canis familiaris *(dog)	**ENSCAFT00000015169 **(g, pa)
*Dasypus novemcinctus *(nine-banded armadillo)	**ENSDNOT00000001508 **(g, pa)

Accession numbers of new entries are highlighted by "ne". Sequences that have been used for PAML analyses are labeled with "pa".

**Figure 1:**
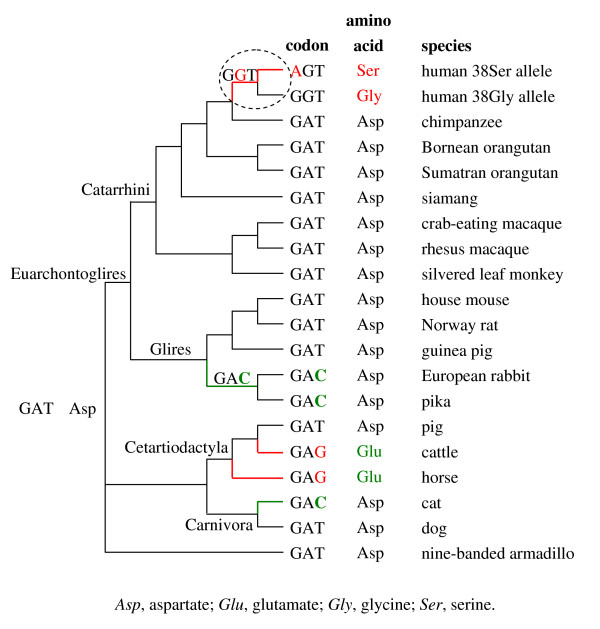
**Remarkable variability at codon 38 of human *KCNE1***. Extant and baseml-reconstructed tripletts (and the encoded amino acids) were mapped onto the widely accepted phylogeny among catarrhine primates [49] and Eutheria [47,48]. The discontinuous line encircles branches defined as foreground in branch-site analysis (codeml model A). Red indicates non-synonymous nucleotide substitutions and radical amino acid replacements; green highlights synonymous nucleotide substitutions and conservative amino acid exchanges. Only newly arisen tripletts are labelled along the internal branches.

In line with the overall prevalence of GAT (Asp) at the site corresponding to codon 38 in human *KCNE1*, baseml suggests GAT as the ancestral character state at the base of the tree (see Figure 1). Mapping the extant as well as the ancestral character states onto the phylogeny in Figure 1 suggests that only few evolutionary steps occurred since the last common ancestor of the studied species. Thus, two independent synonymous substitutions from GAT to GAC can be assumed for the terminal branch to cat and for the internal branch uniting pika and European rabbit. Moreover, the cattle and horse branches are characterized by a parallel non-synonymous substitution changing the coding from GAT to GAG. However, the associated exchange from Asp to Glu is conservative since both amino acids are negatively charged (for amino acid classification see, e.g. [19]). The functional consequences of the Asp/Glu shift can thus be regarded as neglegible.

The human stem lineage represents the only exception from the wide phylogenetic conservation of codon 38 and its counterparts (Figure 1). According to the presence of the Gly38Ser polymorphism in all human populations studied (e.g. [9,11,12,14-16] and present study), two non-synonymous transitions can be assumed for the branch leading to modern humans: 1) a change from GAT to GGT, and, thus, from Asp to glycine (Gly), and 2) a shift from GGT to AGT, which corresponds to a Gly to Ser replacement at the amino acid level (Figure 1). Both, the Asp/Gly shift (negatively charged/hydrophobic) and the Gly/Ser shift (hydrophobic/polar) are moderately radical. The moderately radical character of the amino acid exchanges argues for their effectiveness without disrupting protein function (for amino acid classification see, e.g. [19]). In accordance with the human-specificity of the Ser38Gly polymorphism, branch-site analysis (codeml) provides highly significant support (*p*-value = 0.01) for the branch-site test of positive selection across the human foreground. Not surprisingly, codon 38 of human *KCNE1 *belongs to the specific site class (*p*-value = 0.970) that is under positive selection across the human foreground (ω_2 _= 187), but is under negative selection (ω_2*a *_= 0.073) or neutral evolution (ω_2*b *_= 1) across the remaining phylogeny (Table 5; Figure 1). The other codon sites belonging to this specific site class are codons 105 and 115. The high ω-value assigned to the specific site class (= 187) most likely results from the short branch leading to modern humans. Irrespectively, there is no doubt about the nature of the selective force acting particularly on codon 38 of human *KCNE1*, i.e. strong positive Darwinian selection (Figure 1).

**Table 5:** Parameter estimates from modified branch-site model A [50]

Site class	Proportion	Remaining phylogeny ω	Human foreground ω
0	0.709	0.073	0.073
1	0.267	1.000	1.000
2a	0.017	0.073	187
2b	0.006	1.000	187

Codon sites pinpointed as positively selected across the human foreground: 38 (*p*-value = 0.970), 105 (*p*-value = 0.686), and 115 (*p*-value = 0.962).

### Motif search and Western blotting

**Figure 2:**
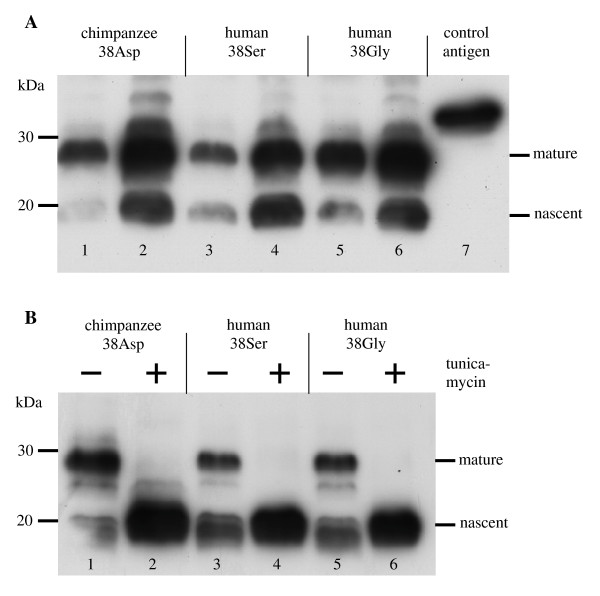
**KCNE1 proteins from chimpanzee and human are highly N-glycosylated**. **A **HEK293 cells were transfected with 1 μg (lanes 1, 3, and 5) or 3 μg (lanes 2, 4, and 6) expression plasmids for the indicated KCNE1 variants. Nascent (n) proteins and mature (m) proteins were detected by Western blotting. **B **HEK293 cells transfected with 1 μg expression plasmids were treated without (lanes 1, 3, and 5) and with tunicamycin (lanes 2, 4, and 6). Addition of the N-glycosylation inhibitor tunicamycin results in loss of the glycosylated KCNE1 variants. **A, B **15 μg total protein were loaded per lane.

According to results from PROSITE motif search the human 38Gly variant is characterized by a glycosaminoglycan attachment motif (SerGlyXGly) spanning from amino acid positions 37–40 that is absent in the 38Ser variant and the non-human KCNE1 orthologs. Motiv search moreover found motivs for N-glycosylation, protein kinase C phosphorylation, and N-myristoylation offside the glycosaminoglycan attachment motif (not shown). To adress the question whether the evolution of the Gly38Ser polymorphism in humans entailed differences in glycosylation, we expressed different KCNE1 variants in human HEK293 cells and analysed the KCNE1 proteins by Western blotting (Figure 2A and 2B). Beside the 15 kDa KCNE1 proteins, most probably representing the nascent forms (Figure 2A, n) we could also detect slower migrating KCNE1 proteins, representing the mature, posttranslationally modified proteins (Figure 2A, m). The mobility shift resulted from N-glycosylation as addition of the N-glycosylation inhibitor tunicamycin resulted in complete loss of slower migrating KCNE1 variants (Figure 2B, lanes 2, 4 and 6). However, both human variants and the chimpanzee ortholog of KCNE1 (representing the ancestral 38Asp situation) revealed no obvious differences in mobility. This shows that all KCNE1 variants are highly N-glycosylated and suggests that the 38Gly variant does not confer an extra glucosaminoglycan modification. Therefore, we conclude that the presumed functional differences linked to the specific evolution of *KCNE1 *codon 38 in humans are not due to differences in posttranslational modification. Instead, they seem to be caused exclusively by the moderately radical character of the involved amino acid exchanges (Asp > Gly, Gly > Ser).

## Discussion

### Deviating allelic copy numbers in one heterozygote Gly38Ser brain sample

As outlined above, we consider the presented pyrosequencing results as very reliable, because all gDNA based runs confirmed the genotypes inferred from direct sequencing. This applies particularly to brain sample 11 whose deviating allelic copy numbers had even been validated by a total of eight measurements. It can thus be excluded that technical problems account for the about 20:80 distribution of 38Ser- and 38Gly-coding alleles in the gDNA of brain sample 11. Accumulating experimental evidence suggests that neural progenitor cells often display loss and/or gain of chromosomes. Consequently, a considerable percentage of aneuploid neurons populate the mature brain [20]. Chromosome painting studies, for instance, revealed approximately 4% chromosome 21 aneuploid cells (both postmitotic neurons and non-neural cells) in normal adult human brain [21]. These aneuploid neurons are projected into the appropriate anatomical areas and appear to be functionally active [22]. In this light, genetic mosaicism must be considered a frequently observable feature of the human brain. The most likely explanation for the uneven distribution of chromosome 21 alleles in brain sample 11 is gain and/or loss of a chromosome 21 copy in a relatively high proportion of cells in the dissected cortex area. Another possibility could be a uniparental disomy for chromosome 21 (UPD21), since the sampled man was phenotypically normal, what is a general feature of UPD21 [23,24]. However, as the question for the underlying mechanism is second ranked in the present context we did not further investigate the alternatives herein.

### Sporadic deficiency of 38Ser-coding mRNAs in heterozygote humans

Four different mechanisms could principally explain the observed absence of 38Ser-coding alleles in cDNAs from three individuals with a heterozygous genotype (two lymphoblastoid cell lines and brain sample 11): i) monoallelic expression of the 38Gly-coding allele as a consequence of null mutations in the regulatory region of the 38Ser-coding allele, ii) allelic dropout of the 38Ser-coding allele, iii) monoallelic expression of the 38Gly-coding allele due to silencing of the 38Ser-coding allele by genomic imprinting, and iv) genomic recoding of 38Ser-coding transcripts to 38Gly-coding alleles by A to I editing. We consider it very unlikely that the observed pattern results from deleterious mutations in the regulatory region since null mutations should affect not only the 38Ser-coding allele. Allelic dropout can further be ruled out as the 38Ser allele did not drop out when analysing the other 21 heterozygote samples. One possible explanation for the deviating allele frequencies in the three genomically heterozygous individuals is genomic imprinting, even though *KCNE1 *does not belong to the known imprinted genes, neither in humans nor in mouse [25,26]. Alternatively, genomic recoding at the mRNA level might account for the observed pattern. According to this, an ADAR sporadically recodes the adenosine at *KCNE1 *codon 38 (AGU) to inosine (IGU), which is equivalent to guanosine (GGU) for the translational machinery [17]. This assumption is additionally corroborated by a hint for A to I editing at *KCNE1 *codon 38 in GenBank entry BC046224 ("G in cDNA is A in the human genome" [27]). If one further considers evidence for genomic recoding of the human potassium voltage-gated channel, shaker-related subfamily, member 1 (KCNA1) [28] as well as of ion channels in diverse non-primate taxa such as rodents [29,30], insects [31,32], and molluscs [33,34], genomic recoding might indeed be the reason for the observed specific allele frequencies in three humane heterozygotes. Future investigations will finally clarify whether genomic imprinting or genomic recoding are causative for the sporadic occurence of deviating allele frequencies in the gDNA/cDNA comparison in humans. Irrespectively, the present data provide first time evidence for the coincidence of positive selection at a polymorph codon site and the sporadic absence of one allele in the mRNA of heterozygotes.

### Functional considerations in the light of *KCNE1 *variance and divergence

The finding that the Gly38Ser polymorphism is a human-specific feature that emerged under positive selection poses the question for its adaptive value (see Figure 1). Given the relevance of IKs channels for inner ear homeostasis and the function of muscles and neurons it is tempting to speculate that the evolution of the Gly38Ser polymorphism is connected to the physiological challenges of specific human features such as speech perception, upright and enduring walking, and advanced cognitive capabilities. Regardless of these speculations, the evolution of the human-specific Gly38Ser polymorphism probably conferred some functional advantage at the cellular level. This conclusion agrees well with the results of a recent study showing an involvement of the Gly38Ser polymorphism in the fine-tuning of IKs channels [35]. As we found no evidence for changes in glycosylation between the chimpanzee protein and the two human KCNE1 variants (see Figure 2), the functional relevance of the Gly38Ser polymorphism most probably results from the moderately radical character of the amino acid exchanges involved (Asp > Gly, Gly > Ser). While amino acid position 38 belongs to the extracellular N-terminus of human KCNE1, the two other amino acid sites pinpointed herein as positively selected (105 and 115) belong to the intracellular C-terminus [1]. As such they might be involved in the interaction with KCNQ1, namely in the regulation of channel assembly, open-state destabilization, and kinetics of channel deactivation [36].

According to the population genetic data a subtle heterozygote advantage accounts for the maintenance of the Gly38Ser polymorphism in humans. However, the extent of such an heterozygote advantage might depend on the environment, since we found a significant heterozygote excess solely in Yoruba from Nigeria, but nearly no differences between the observed and expected genotype counts in Japanese from Tokyo (see Table 3). The depletion of 38Ser homozygotes in the Yoruban sample and particularly the sporadic absence of 38Ser-coding mRNAs in Central Europeans further suggest that the expression of the 38Ser allele is disadvantageous under certain conditions. 38Ser to 38Gly recoding or monoallelic expression of the 38Gly allele might then represent mechanisms to be functionally homozygote 38Gly when required by specific physiological conditions. Beyond this, there might be individual differences in the capability of genomic recoding or genomic imprinting. Such a scenario could explain why we found a depletion of 38Ser-coding alleles only in two out of 19 heterozygous cell lines and one out of five heterozygous brain samples.

## Conclusion

It is well established that aberrant editing patterns can lead to diseases such as epilepsy [30], depression [37], amyotrophic lateral sclerosis [38] and malignant gliomas [39]. Likewise, genomic imprinting is assumed to play a role in the susceptibility to diverse diseases [40,41]. In extension of this, individual differences in the capability of suppressing the expression of the 38Ser variant might predispose 38Ser carriers more or less to LQTS. For instance, sex-specific differences in the depletion of 38Ser-coding mRNAs could explain why the Gly38Ser polymorphism correlates significantly with QT length in men from North Israel, but not in women [11]. Furthermore, sporadic 38Ser deficiency might have obscured the recognition of an associations between Gly38Ser and LQTS in diverse Central European cohorts (see [9,14-16]). However, these are speculations since it remains an open question whether the present conclusions drawn from lymphoblastoid cell lines and brain samples can be transferred to heart tissue. Provided that future investigations will confirm a sporadic depletion of 38Ser- coding mRNAs in heart of Gly38Ser heterozygotes and 38Ser homozygotes, this would underline the relevance of mRNA data in future association studies between Gly38Ser variant and LQTS. For apparent reasons heart tissue can only be collected post mortem. Another important question would thus be if peripheral blood lymphocytes reflect the recoding or imprinting pattern in heart tissue. Only under such circumstances peripheral blood lymphocytes could be taken as a predictor for the situation in heart. Irrespective of these considerations, the present evolutionary approach reveals an unusual, if not unique, pattern at the codon level, i.e. coincidence of positive Darwinian selection and polymorphism with a sporadically suppressed expression of one allele. This further corroborates the previously demonstrated functional relevance of the Gly38Ser polymorphism of KCNE1 [35].

## Methods

### Human sample

The present sample comprised lymphoblastoid cell lines of 29 individuals and brain samples (frontal cortex) of another 14 individuals. All individuals were Central Europeans. Anonymized brain samples were collected during pathomorphological diagnostics and immediately shock-frozen in liquid nitrogene. Lymphoblastoid cell lines were generated by Epstein-Barr virus transformation of peripheral blood leucocytes propagated according to standard procedures [42]. We extracted gDNAs using standard procedures. Total RNAs were extracted using TRIzol Reagent (Invitrogen). cDNAs were synthesized from total RNAs with random primers and SuperScript III (Invitrogen) following the supplier's protocol. Use of the brain samples was in accordance with the Helsinki Declaration and approved by the local ethics committee [Aerztekammer Rheinland-Pfalz, Decisions 837.103.04 (4261) and 837.073.07 (5608)].

### Direct sequencing of human samples

For direct sequencing of gDNAs, PCR was carried out using FastStart Taq DNA Polymerase (Roche) and normal hot start PCR. The primers used bound to exon 3, in particular -18 and +26 bp upstream and downstream of start and stop codon, respectively (forward primer: 5'-TGCCTGGGAAGTTTGAGCTG-3'; reverse primer: 5'-CAGGTTGCCAGGCAGGATG-3'). PCR conditions were as follows: 2 min, 94°C; 35× (40 s at 94°C, 40 s at 65°C, 40 s at 72°C); 5 min, 72°C. Following exonuclease I and shrimp alkaline phosphatase digestion, dye terminator cycle sequencing of the PCR products was performed using the PCR primers and the CEQ DTCS Quick Start Kit (Beckman Coulter). Sequencing products were separated on a Beckman Coulter CEQ 8000 Genetic Analysis System. SCF-files were analyzed by visual inspection using BioEdit [43].

### Quantitative Allele-Specific Expression Analysis of human samples

To more accurately measure the A versus G allele ratios of the A112G (Gly38Ser) variant in gDNAs and cDNAs, Quantification of Allele-Specific Expression by Pyrosequencing (QUASEP) was used [44]. QUASEP allows to accurately quantify the relative amount of one allele to the other, if their sequences differ in at least one base pair. The primer sequences for QUASEP were designed using the Pyrosequencing Assay Design Software (Biotage AB).

PCR products for QUASEP-analyses of the *KCNE1*-A/G-SNP rs1805127 were generated from gDNAs and cDNAs of lymphoblastoid cell lines and brain samples using FastStart Taq DNA Polymerase (Roche) and normal hot start PCR with the forward primer 5'-AGAGGGCCTCCAGCTTGC-3' and 5' biotinylated reverse primer 5'-GCAGGGTGGCAACATGTC-3' according to standard protocols. Pyrosequencing was performed on a PSQ™96MA Pyrosequencing System (Biotage) with the PyroGold SQA reagent kit (Biotage) using the forward sequencing primer 5'-CCAGCTTGCCGTCAC-3'. The PSQ™96MA 2.1.1 software (Biotage) was used for data analysis.

In order to assess whether RNA extraction or specific conditions of some of the samples affect the results of QUASEP-analyses of the *KCNE1*-SNP rs1805127 we performed analogue QUASEP-analyses of the highly heterozygous A/G-SNP rs11254413 of the housekeeping gene *TRDMT1*. Hot start PCR was carried out using the forward primer 5'-TGGCTATCCTCTACAAAATGACAA-3' and 5' biotinylated reverse primer 5'-CGGCAGGGTGATATGACTGAT-3'. For pyrosequencing we took the forward sequencing primer 5'-CCTTGGGAGAATATCTAGAA-3'. QUASEP of *TRDMT1*-SNP rs11254413 was confined to gDNAs and cDNAs of lymphoblastoid cell lines.

### Interspecies comparison

To assess the evolution of *KCNE1 *across Eutheria, we complemented the human *KCNE1 *data with gDNA and cDNA data from non-human species (Mammalia, Eutheria), using NCBI and ENSEMBL databases. New sequences were generated from four non-human primates, i.e Bornean orangutan, siamang, crab-eating macaque, and silvered leaf monkey. Total RNA was extracted from testis (crab-eating macaque) and lymphoblastoid cell lines (all others) using TRIzol Reagent (Invitrogen). cDNAs were synthesized from total RNA with SuperScript II reverse transcriptase (Invitrogen), using the reverse gene-specific primer 5'-TCAGGTTGCCAGGCAGGAT-3'. The target cDNA encompassing the entire KCNE1-coding sequence was amplified with the forward gene-specific primer 5'-AGCCAAGGATATTCAGAGGT-3' and the reverse gene-specific primer mentioned above by standard PCR under the following conditions: 3 min at 94°C, 30 × (30 s at 94°C, 30 s at 58°C, 40 s at 72°C), 5 min at 72°C. Direct sequencing was carried out as described for the human samples. In a final step, we completed the dataset with GenBank entries from another 14 eutherian representatives, thus generating a dataset comprising orthologs of human and 18 non-human species (see bold accession numbers in Table 4). The sequences were translated, ClustalW aligned, and re-translated using BioEdit [43]. The non-human sequences were moreover scrutinized for polymorphisms of the codon site ortholog to human *KCNE1 *38, using ENSEMBL data. We finally checked the respective codon site of non-human *KCNE1 *sequences for hints of genomic recoding at the mRNA level. Therefore, we compared gDNAs retrieved from ENSEMBL and NCBI with the actual set of cDNAs deposited in the Nucleotide collection and EST databases at NCBI (see Table 4).

### Statistical analyses

Population genetic analyses were carried out using allele frequencies and genotype counts of the geographically distinct human samples covered by HapMap phase II (120 Central Europeans, 90 Han Chinese from Beijing, 88 Japanese from Tokyo, 120 Yoruba from Ibadan/Nigeria). Expected genotype counts were calculated using Fstat 2.9.3 [45]. We engaged the same software to infer the fixation index, Fis. Fis is a measure of the heterozygote deficit within subpopulations. Consequently, positive Fis-values point to a heterozygote deficit, while negative Fis-values indicate a heterozygote excess. Fstat has moreover been used to test for deviations from Hardy-Weinberg equilibrium (400 randomisations). We corrected for multiple testing by strict Bonferroni adjustment, thus lowering the 5% level of significance to 0.0125.

The evolutionary history of the codon position corresponding to codon 38 in human *KCNE1 *was traced back using ancestral sequences reconstructed by baseml (PAML 3.15 [46]). The dataset comprised the non-human *KCNE1 *orthologs plus the two human alleles. The intree used is shown in Figure 1 and represents the commonly accepted phylogeny among mammals [47,48] and primates [49].

We furthermore assessed the evolutionary regime acting on *KCNE1 *as a whole using the ratio of non-synonymous (amino acid altering) to synonymous (silent) nucleotide substitution rates (= *dn*/*ds *= ω). Rate ratios > 1 are commonly accepted as a conservative measure of positive selection (= adaptive evolution) while rate ratios = 1 and < 1 are indicative of neutral evolution and negative selection, respectively. To study the evolution of *KCNE1 *across the human branches (foreground, see Figure 1) in comparison to the remaining phylogeny, we employed the modified version of branch-site model A [50] of codeml (PAML 4 [46]). Model A assumes four site classes: site class 0 integrates codon sites that are conserved throughout the entire phylogeny (foreground plus remaining phylogeny) with 0 < ω_0 _< 1. Site class 1 comprises codon positions that evolve neutrally throughout the entire phylogeny with ω_1 _= 1. Site classes 2a and 2b include codon sites that undergo positive selection on the foreground, but are under negative selection or neutral evolution across the remaining phylogeny. The model involves four free parameters: the proportion (p_0_) and the ω estimate (ω_0_) of site class 0; the proportion estimate of site class 1 (p_1_) and the ω estimate of the codon sites that evolve under positive selection across the foreground (ω_2_). The codon frequency was estimated from a 3 × 4 contingency table. Ambiguity data were not removed from the data (cleandata = 0). The Bayes empirical Bayes approach was used to identify codon sites under positive selection along the (human) foreground branches [51]. To test for the significance of the results, the branch-site test of positive selection (test 2 in [50]) was carried out. Therefore, we compared twice the log likelihood difference between model A with ω_2 _> 1, estimated from the data, and model A with ω_2 _= 1 fixed with the critical values from chi-square distribution with 1 degree of freedom.

### Motif search

Motif search was carried out using the PROSITE database as implemented in the PredictProtein server [52].

### Expression of *KCNE1 *in HEK293 cells and Western blotting

HEK293 cells (ATCC CRL 1573) were grown at 37°C under 5% CO_2 _in Dulbecco's Modified Eagle Medium (Gibco) supplemented with 10% fetal calf serum. 2.5 * 10^5 ^cells were transfected in six well plates (Greiner) with 1 μg or 3 μg of expression plasmids pcDNA3.1-chimpanzee_*KCNE1*_GAT, pcDNA3.1-human_*KCNE1*_AGT or pcDNA3.1-human_*KCNE1*_GGT by calcium phosphate coprecipitation. To assess the extent of N-glycosylation, tunicamycin (Sigma) was added to the medium to a concentration of 5 μg/ml 10 hours after transfection. For Western blotting, whole cell lysates were prepared 24 hours after transfection. Cells were washed three times in phosphate-buffered saline and pelleted by centrifugation at 300 g. The pellet was resuspended in 5 volumes of ice-cold NP-40 (Fluka) lysis buffer consisting of 137 mM KCl, 20 mM HEPES pH 7.9, 1% Nonidet P-40, 5 mM NaF, 10 μg/ml aprotinin, 10 μg/ml leupeptin, 1 mM dithiothreitol, 0.5 mM phenylmethylsulfonyl fluoride, and incubated on ice for 40 min. The lysate was cleared by centrifugation at 80,000 g for 30 min. Protein concentrations were determined using the Bradford method (Bio-Rad). Cell lysates were separated by SDS-PAGE and proteins were transferred to polyvinylidene difluoride membranes (Millipore) for 20 min at 150 mA, using a Tris-glycine buffer system. After blocking, membranes were incubated with the primary antibody directed against KCNE1 (Calbiochem). As secondary antibody, a 1:5,000 dilution of peroxidase-conjugated donkey, anti-rabbit antibody (GE healthcare) was used. The control antigen corresponds to residues 67 to 129 of human KCNE1 fused to glutathione S-transferase (Calbiochem).

### Sequence data

New sequences reported in this manuscript have been submitted to GenBank under accession numbers EF514881 to EF514888.

## Abbreviations

cDNA: complementary DNA; dn/ds = ω: ratio of non-synonymous (amino acid altering) to synonymous (silent) nucleotide substitution rates; gDNA: genomic DNA; IKs: slowly activating delayed rectifier potassium channel; KCNA1: potassium voltage-gated channel, shaker-related subfamily, member 1; KCNE1: potassium voltage-gated channel, Isk-related family, member 1; KCNQ1: potassium voltage-gated channel, KQT-like subfamily, member 1; LQTS: long-QT syndrome; SNP: single nucleotide polymorphism; TRDMT1: tRNA aspartic acid methyltransferase 1; UPD21: uniparental disomy for chromosome 21.

## Authors' contributions

HH and HZ initiated the study on the basis of preliminary data. HH contributed to the laboratory work, carried out databank mining, performed evolutionary and population genetic analyses, and wrote and edited the manuscript. UZ and TH provided human and non-human samples, and carried out direct and pyrosequencing. FO conducted HEK293-expression of KCNE1 and subsequent protein work. AP contributed to the population genetic analyses. UZ, FO, AP, HZ, and TH participated in the data interpretation and contributed to the writing of the manuscript. The experimental design was jointly conceived by all of the authors. All authors read and approved the final manuscript.
